# Studies on the Dual Cytotoxicity and Antioxidant Properties of *Berberis vulgaris* Extracts and Its Main Constituent Berberine

**DOI:** 10.1155/2018/3018498

**Published:** 2018-01-08

**Authors:** Lamyae El khalki, Mounir Tilaoui, Abdeslam Jaafari, Hassan Ait Mouse, Abdelmajid Zyad

**Affiliations:** Laboratory of Biological Engineering, Natural Substances, Cellular and Molecular Immuno-Pharmacology Team, Immunobiology of Cancer Cells, Faculty of Sciences and Technology of Beni Mellal, P.O. Box 523, 23000 Beni-Mellal, Morocco

## Abstract

The present study attempts to investigate the cytotoxic activity of ethanol and ethyl acetate extracts of the Moroccan *Berberis vulgaris* and its major component berberine, together with exploring their antioxidant properties. It also consists of studying the combination effect of berberine and S-nitroso-N-acetylpenicillamine (SNAP), a nitric oxide (NO) donor, against the human breast adenocarcinoma cell line (MCF-7). Using the MTT assay, we report a differential cytotoxic effect of ethanol and ethyl acetate extracts since the ethanol extract is more cytotoxic than the ethyl acetate one, with IC_50_ = 3.54 μg/mL and 596.71 μg/mL, respectively. Interestingly, no cytotoxic effect was observed against normal cells. Furthermore, these extracts showed a remarkable antioxidant activity as measured by the DPPH free radicals scavenging assay. In fact, the IC_50_ values are 69.65 μg/mL and 77.75 μg/mL for the ethanol and ethyl acetate extracts, respectively. In addition, several concentrations of berberine, when combined with the NO donor used at IC_30_, induced a synergistic cytotoxic activity at concentrations ranging from 8.40 μM to 33.60 μM, as revealed by the combination index values, using the Chou–Talalay method. However, at the other concentrations tested, an antagonistic effect was observed. The observed cytotoxicity was related to apoptosis induction as demonstrated by the annexin-V-streptavidin FITC-staining analysis.

## 1. Introduction

Our knowledge of plants and their benefits is as old as mankind. Man discovered very early the therapeutic properties of certain plants to overcome his suffering and improve his health. Thus, we chose to work on *Berberis vulgaris* from Oujda, east of Morocco, a plant of the Berberidaceae family locally named “Aghriss,” “Izergui,” and “Bou-Semmane” and used in traditional medicine for its antipyretic, hepatoprotective, and anti-inflammatory properties [1]. Several studies have been conducted on its biological activities and more specifically on berberine, an isoquinoline alkaloid, considered as an active molecule with many properties such as hypoglycemic, antibacterial, antifungal, anti HCV, and anticancer activities [2–8]. Indeed, despite the significant advances of modern medicine, we always note a slight failure of conventional drug treatments in the case of high incidence of side effects and development of resistance. Therefore, the first part of this work consists of comparing the cytotoxic activity of *Berberis vulgaris* extracts against breast cancer cells and normal human cells, as the nonselectivity of chemotherapy treatment is what causes the systemic toxicity. We also investigated the molecular mechanisms of the cytotoxicity, as recent knowledge on molecular carcinogenesis has provided the potential for therapeutic intervention in cancer by specifically targeting and sensitising cancer cells to apoptosis [9].

In the second part, this work aims to explore the combination effect of berberine and S-nitroso-N-acetylpenicillamine (SNAP), a nitric oxide (NO) donor, against breast cancer cells. Knowing that NO is a free radical synthesised from L-arginine by NO synthase (NOS), three isoforms of NOS (neuronal (nNOS), endothelial NOS (eNOS), and inducible NOS (iNOS)) are expressed in various tissues and cells. NO plays an important role in different biological responses, including the regulation of vascular tone, neurotransmission, antiviral defence, and immune responses [10]. Recent studies have also demonstrated that NO is an interesting regulator of cell death [11]. This part of the study was designed to explore the effect of NO on the viability of breast cancer cells, alone and in combination with berberine.

## 2. Materials and Methods

### 2.1. *Berberis vulgaris* and Its Extracts

The plant used in this study was collected at the end of autumn to early winter (December 2014), in the region of Oujda, east of Morocco. The part used in this study is the root barks of the plant. The root barks were isolated and dried in the shade at room temperature and then crushed and ground. Then, the extraction was performed with a Soxhlet extractor using two types of solvents of different polarities: ethyl acetate and ethanol. The extracts obtained were concentrated using a rotary evaporator until the total evaporation of the solvent.

### 2.2. Chemicals

Dulbecco's modified Eagle's medium (DMEM), dimethyl sulfoxide (DMSO), ethylenediaminetetraacetic acid (EDTA), phosphate-buffered saline (PBS), methyl tetrazolium (MTT), crystal violet, ethyl acetate, ethanol, methanol, trifluoroacetic acid and acetonitrile (HPLC–MS grade), Ficoll, isopropanol, hydrochloric acid (HCl), sodium dodecyl sulfate (SDS), annexin-V-FITC, trypan blue, S-nitroso-N-acetylpenicillamine (SNAP), berberine, and cisplatine were purchased from Sigma-Aldrich (Saint Quentin, France).

### 2.3. Cell Line and Culture

The tumour cell line used in this study was the human breast adenocarcinoma (MCF-7) generously provided to our laboratory by the Gustave Roussy Institute (Villejuif, France). These cells are cultured at 37°C in a humidified atmosphere with 5% CO_2_ in a culture medium (DMEM) supplemented with 5% of fetal bovine serum, 100 UI/mL of penicillin, 100 *μ*g/mL streptomycin, and 0.2% sodium bicarbonate.

### 2.4. Cytotoxicity Measurement

The cytotoxic activity was studied against the MCF-7 tumour cell line using the colourimetric methyl tetrazolium (MTT) test as described by Mosmann [12] and modified in our laboratory [13]. The target cells were washed twice and placed on 96-well microtiter plates (Bioster, Italy) at a density of 3 × 10^4^ cells/mL in 100 *μ*l/well of the completed culture medium. Then, 100 *μ*l of the culture medium, containing decreasing doses of the ethanol or ethyl acetate extracts already solubilised in the DMSO, was added in each well of the microtiter plates containing cells for a final volume of 200 μl, with 400 μg/mL as the initial concentration, to reach a final concentration of 0.78 μg/mL. The cisplatine was used as a positive control. After exposure of the cells to serial concentrations of tested products for 48 h at 37°C and 5% CO_2_, 100 *μ*l of medium was carefully aspirated from each well and replaced by 20 *μ*l of MTT solution (5 mg/mL of PBS). After incubation in the same conditions for 3 h, the plates were treated with 100 μl of isopropanol/HCL (10 mL: 2.5 μl) solution to dissolve the blue intracellular formazan product. 30 min later at 37°C, the solubilised formazan produced by the metabolically active cells was measured by scanning the 96-well plates at dual wavelength of 540–630 nm using a Multiskan apparatus (Labsystems, Helsinki, Finland). Thus, the cytotoxic effect was calculated using the following formula:

(1) $$\begin{array}{c} \text{\% Cell viability}=\left(\frac{\text{OD}}{{\text{OD}}_{0}}\right)*100, \end{array}$$

where OD_0_ and OD are the optical density obtained, respectively, for the negative control cells receiving DMSO (0.5%) alone and the ethanol extract- or ethyl acetate extract-treated cells. Three independent sets of experiments performed in duplicate were evaluated.

### 2.5. Cytotoxic Effect against Human Peripheral Blood Mononuclear Cells (PBMCs)

This test was realised in order to evaluate the effect of the tested extracts on normal human cells. To isolate the PBMCs, blood samples (10 mL) in sterile heparinised tubes were collected under medical and ethical committee control from healthy volunteer donors.

Peripheral blood mononuclear cells were isolated using standard Ficoll-hypaque density centrifugation. The interface lymphocytes were harvested and washed twice with sterile phosphate-buffered saline (PBS). The cytotoxic effect was measured by the MTT test in the same conditions and concentrations as detailed above for the tumour cells.

### 2.6. Chemical Analysis of *Berberis vulgaris* Extracts

This analysis was conducted by a chromatography coupled to a mass spectrometry (HPLC-MS) of the National Centre for Scientific and Technical Research (CNRST) laboratories (Rabat, Morocco). The HPLC-MS analysis was conducted at 279 nm and 30°C using an RP-C18 column (150 × 4.6) × 5 μm, with a Thermo Fisher apparatus equipped with a surveyor pump coupled to the PDA detector (diode array detector: 200–600 nm) and a mass spectrometry-ion trap LCQ advantage (ESI) (Thermo Finnigan, San Jose, CA, USA). A constant flow rate of 0.5 mL/min of a polar mobile phase (Solution 1: water/0.05% trifluoroacetic acid; Solution 2: acetonitrile/0.05% trifluoroacetic acid) polarity changed during 76 min of the analysis, pushing 20 *μ*l of the extract to be analyzed in an apolar stationary phase column. The sample passed directly to a mass spectrometer where its principle resided in the separation in the gas phase-charged molecules (ions) based on their mass/charge ratio *m/z*. The full scan mass data *m/z* were obtained in both positive and negative modes.

### 2.7. Analysis of Apoptosis Induction

The apoptosis analysis was performed using the annexin V-streptavidin FITC test. Briefly, MCF-7 cells (4 × 10^4^ cells) were treated in the 24-well plates with the IC_30_ of ethanol extract (0.2 μg/mL) or the IC_30_ of berberine (0.67 μg/mL) or grown under serum starvation conditions (used as a positive control). After 6 h, 12 h, and 24 h incubations in the same culture conditions as the above, the cells were washed with PBS, stained with annexin V-fluorescein isothiocyanate (FITC), and treated with streptavidin conjugated to FITC. The fluorescence was visualised using an Olympus BX51 microscope equipped with fluorescence filter and CytoVision software in order to detect apoptosis induction. The assay is based on the ability of annexin V (green fluorescence) to bind to phosphatidylserine exposed on the surface of cells undergoing apoptosis [14].

### 2.8. Antioxidant Activity Measurement

The studied extracts and berberine were diluted in methanol, and 150 *μ*l of 0.004% DPPH was added in each well containing the serial concentrations of extracts, berberine, and the positive control (vitamin C). After incubation in darkness at room temperature for 30 min, the absorbance was measured at 515 nm [15]. The presence of DPPH radicals resulted in a dark purple colour of the solution. Reducing DPPH radicals by an antioxidant results in the discolouration of the solution. The percentage of inhibition is calculated using the following equation:

(2) $$\begin{array}{c} \text{\% Inhibition}=100*\left(\frac{\left(A_{0}-A_{s}\right)}{A_{0}}\right), \end{array}$$

where at 515 nm wavelength *A*_0_ is positive control absorbance and *A*_*S*_ is extracts and berberine absorbance.

### 2.9. Nitric Oxide Effect on the MCF-7 Tumour Cell Line Using the Crystal Violet Test

We used S-nitroso-N-acetylpenicillamine (SNAP) as a nitric oxide (NO) donor since it releases NO under physiological conditions, making it a useful tool for studying the pharmacological and physiological actions of NO [10]. The cytotoxic activity was studied against the MCF-7 tumour cell line and was measured using the crystal violet staining test as described by Zyad et al. [16]. The target cells were washed twice and placed on 96-well microtiter plates (Bioster, Italy) at a density of 6 × 10^3^ cells/mL in 100 *μ*l/well of the completed culture medium. Then, 100 *μ*l of the culture medium containing decreasing concentrations of SNAP, already solubilised in the DMSO and PBS, was added in each well of microtiter plates containing cells for a final volume of 200 μl. After 48 h incubation in a humidified atmosphere at 37°C and 5% CO_2_, the medium was removed and replaced with 100 *μ*l of 0.5% crystal violet solution. After 10 min incubation at room temperature, the plates were washed carefully and viable crystal violet stained cells were lysed with 1% SDS solution. Absorbance at 540 nm was then measured in each well using a Multiskan apparatus (Labsystems, Helsinki, Finland). Thus, the cytotoxic effect was measured using the following formula:

(3) $$\begin{array}{c} \text{\% Cell viability}=\left(\frac{\text{OD}}{{\text{OD}}_{0}}\right)*100, \end{array}$$

where OD_0_ and OD are the optical density obtained, respectively, for untreated cells and SNAP-treated cells. Three independent sets of experiments performed in duplicate were evaluated.

### 2.10. Combination Effect of Nitric Oxide and Berberine against the MCF-7 Cell Line

After measurement of the cytotoxic effect of berberine against the MCF-7 cell line using the crystal violet staining assay, the degree of synergism between berberine and SNAP was determined by using the combination index (CI) analysis at a nonconstant ratio; that is, drug combinations were made by varying the concentrations of one drug (berberine) while keeping the second drug (SNAP) concentration fixed at IC_30_.

The combination effect is measured as described by Chou and Talalay [17] using the following formula:

(4) $$\begin{array}{c} \text{CI}=\frac{D1}{\left(Dx\right)1}+\frac{D2}{\left(Dx\right)2}, \end{array}$$

where *D*1 is dose of drug 1 to produce x% cell death in combination with drug 2, *(Dx)*1 is dose of drug 1 to produce x% cell death alone, *D*2 is dose of drug 2 to produce x% cell death in combination with drug 1, and *(Dx)*2 is dose of drug 2 to produce x% cell death alone. The CI was calculated using Microsoft Excel 2010.

An average CI < 1 indicates synergism, CI > 1 indicates antagonism, and an average CI = 1 indicates additivity effect.

### 2.11. Statistical Analysis

The results are presented in the form of averages ± standard deviation for assays in triplicate. The comparison of the averages is made by Microsoft Office Excel software. The differences are considered significant at *p* < 0.05.

## 3. Results

### 3.1. In Vitro Cytotoxic Effect of *Berberis vulgaris* Extracts

**Figure 1:**
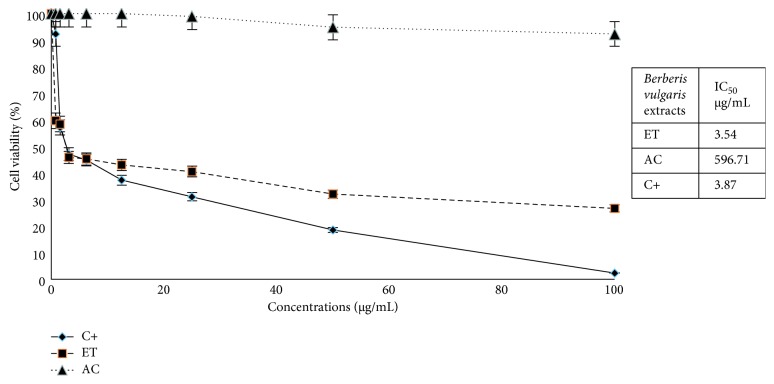
Cytotoxic effect and IC_50_ values of different concentrations of *Berberis vulgaris* extracts against the MCF-7 tumour cell line. ET: ethanol extract, AC: ethyl acetate extract, C+: cis-platin.

The in vitro cytotoxic activity of *Berberis vulgaris* extracts was measured by the MTT assay against the MCF-7 tumour cell line at various concentrations. This activity was evaluated for the ethyl acetate and ethanol extracts of root barks. The results are summarised in Figure 1.

The cells were incubated for 48 h with increasing concentrations of extracts of *Berberis vulgaris*, and the cytotoxic activity was measured by the MTT test as described in the Materials and Methods section. It is shown in this figure that the cytotoxic activity of the various extracts was dose dependent. In fact, as long as the concentration increased, the percentage of cell viability decreased. However, this effect was different from one extract to another; the percentage of viable MCF-7 cells treated by ethanol extract has decreased quickly to only 50% at a concentration of 3.54 μg/mL. However, the ethyl acetate extract was less cytotoxic, with the concentration leading to only 50% of viable population equal to 596.7 μg/mL. It is noteworthy that the cytotoxic effect of the ethanol extract was comparable to that of the positive control (Figure 1).

### 3.2. Evaluation of *Berberis vulgaris* Extract Cytotoxicity against Normal Human Peripheral Blood Mononuclear Cells (PBMCs)

**Figure 2:**
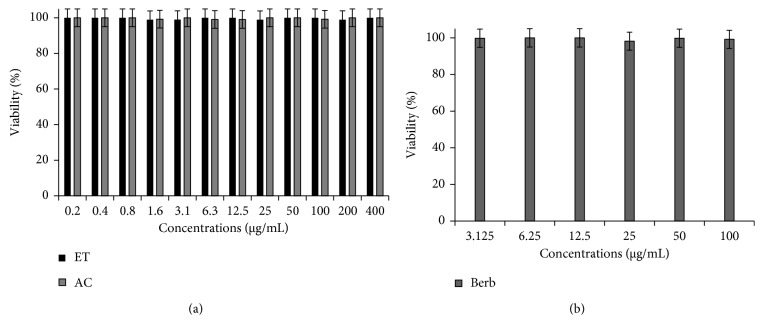
Cytotoxic effect of *Berberis vulgaris* extracts (a) and berberine (b) against normal human cells PBMCs. ET: ethanol extract, AC: ethyl acetate extract, Berb: berberine.

In order to investigate the effect of the ethanol and ethyl acetate extracts and berberine molecule against normal human cells, normal human PBMCs were incubated with increasing concentrations of these extracts in the same conditions as those used against the MCF-7 tumour cells. The obtained results are shown in Figure 2.

The cells were incubated for 48 h with increasing concentrations of extracts of *Berberis vulgaris* and berberine molecule, and then the cytotoxic activity was measured by the MTT test as described in the Materials and Methods section.

This figure shows that despite the increasing concentrations of the stimuli, the viability percentage of PBMCs is substantially constant (almost 100%). The results indicate that these products are tolerated by normal human cells and that the cytotoxicity of studied products is specific for tumour cells. These results are interesting since nontargeting of the tumour cells by actual anticancer products is the origin of their systemic toxicity.

### 3.3. Chemical Composition Analysis of the Ethanol Extract

**Figure 3:**
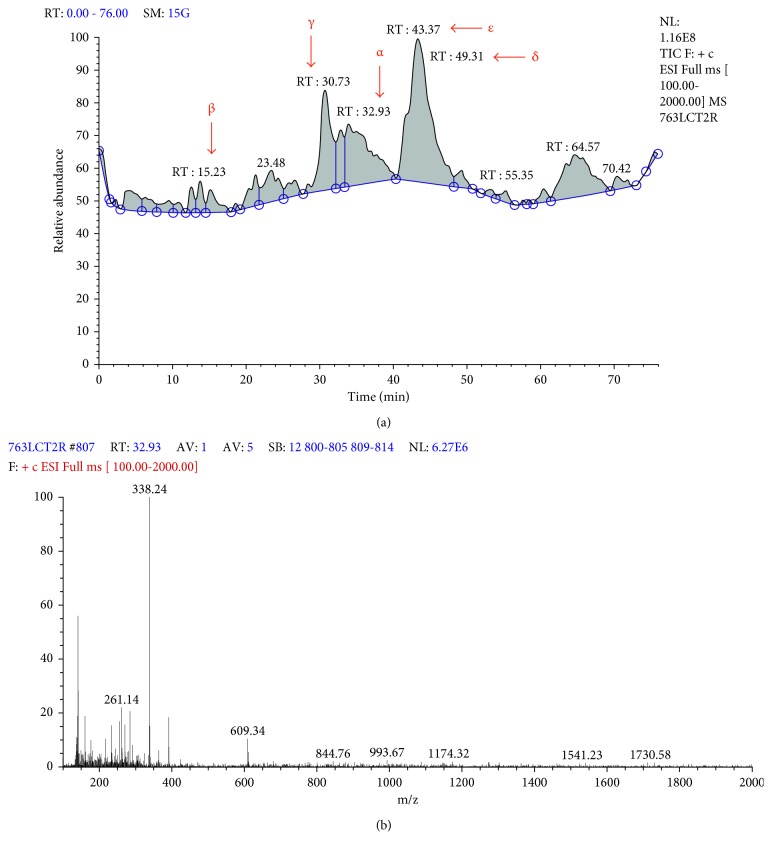
Chemical composition of the ethanol extract of *Berberis vulgaris*. (a) Ethanol extract's chromatogram. (b) Example of the mass spectra of molecule *α* of *Berberis vulgaris* ethanol extract. The molecules are presented as peaks characterised by mass/charge (*m/z*) ratio and retention time.

As long as we found that the ethanol extract of *Berberis vulgaris* had the most cytotoxic effect against the MCF-7 cell line, unlike the ethyl acetate one, we decided to explore the chemical composition of this extract using the high performance liquid chromatography coupled to mass spectrometry (HPLC/MS) method. The obtained results are shown in Figure 3. Given the retention time and the mass/charge (*m/z*) ratio of the various peaks in the chromatogram, we detected five main components: jatrorrhizine, palmatine, columbamine, berberine, and epiberberine, as shown in Table 1.

### 3.4. Cytotoxic Effect of Berberine against the MCF-7 Cell Line

**Figure 4:**
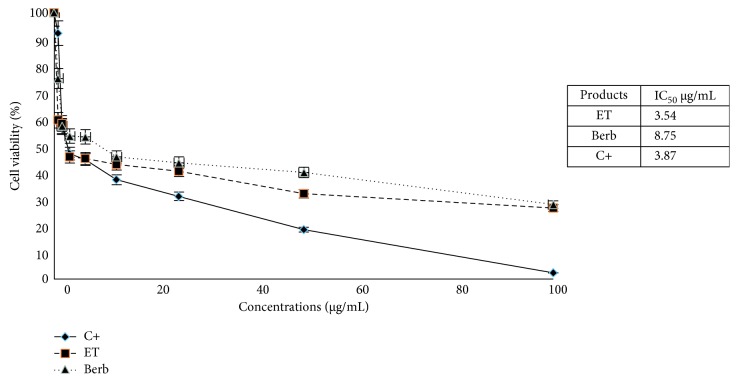
Cytotoxic effect of berberine against the MCF-7 cell line. ET: ethanol extract, Berb: berberine, C+: positive control (cisplatin).

Given that berberine is the most representative molecule in the ethanol extract of *Berberis vulgaris*, we queried whether the cytotoxic activity of this extract was due to this molecule. Then, we conducted a comparison test of the cytotoxic effect of berberine and ethanol extract. The test was carried out using the MTT assay under the same conditions as the previous cytotoxicity tests described above. The results are shown in Figure 4. As shown in this figure, berberine has a similar effect against MCF-7 tumour cells as the ethanol extract with nonsignificant differences, suggesting that the cytotoxic effect of the ethanol extract might be mediated mainly by berberine.

The cells were incubated for 48 h with increasing concentrations of berberine, cisplatin, or ethanol extract of *Berberis vulgaris*, and the cytotoxic activity was measured by the MTT test as described in the Materials and Methods section.

### 3.5. Evaluation of Berberine and Nitric Oxide (NO) Combination Effect against Tumour Cells

Nitric oxide (NO) is a potent antitumour product [23–25]. In order to determine the effect of berberine and NO combination against the MCF-7 tumour cell line (synergy, additivity, or antagonism), we used the combination index (CI) analysis by the Chou–Talalay method. Next, we evaluated the cytotoxic effect of S-nitroso-N-acetylpenicillamine (SNAP) as a NO donor alone and that of berberine alone against the MCF-7 cell line under the same conditions (Table 2). Then, we evaluated the effect of their combination when the SNAP concentration was constant at the IC_30_ values and the berberine concentration varied from 0.525 μM to 1075 μM (Table 3).

It is shown in Table 2 that the cytotoxic activities of NO and berberine alone increased in a dose-dependent manner. In fact, as long as the concentration increased, the cytotoxicity also increased showing a lysis percentage that increased quickly and reached 50% at a concentration of 92.4 μM and 60 μM for SNAP and berberine, respectively.

It is noteworthy that when combined, berberine and SNAP induced a strong cytotoxic activity as it is shown in Table 3. The synergistic activity was observed at concentrations of berberine from 8.4 μM to 33.6 μM combined with the IC30 of SNAP (15.84 μM). However, at high or very low concentrations (<8.4 μM) of berberine, an antagonistic effect was observed (Table 3).

### 3.6. Antioxidant Activity of *Berberis vulgaris* Extracts and Berberine

**Figure 5:**
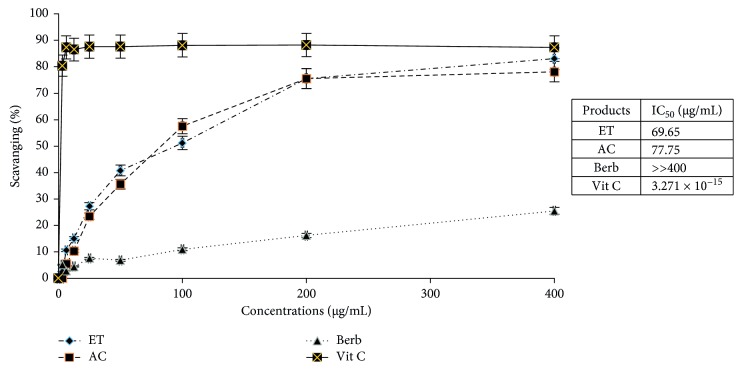
In vitro antioxidant effect of berberine and *Berberis vulgaris* extracts. ET: ethanol extract, AC: ethyl acetate extract, Berb: berberine, Vit C: vitamin C.

To investigate whether berberine and *Berberis vulgaris* extracts show antioxidant activity, we analyzed the percentage of free radical scavenging under the effect of these products using the DPPH (1.1-diphenyl-2-picrylhydrazyl) technique [15]. The results are expressed as a percentage of inhibition of the free radical DPPH (Figure 5).

This figure showed the scavenging percentage of the free radical (DPPH) with increasing concentrations of the studied products (ethanol extract, ethyl acetate extract, and berberine). It is indicated in this figure that as the concentration of these products increased, the trapping percentage of the DPPH also increased. Interestingly, when the ethanol and ethyl acetate extracts showed a comparative antioxidant activity with an IC_50_ = 69.65 μg/mL and 77.75 μg/mL, respectively, with no significant differences, berberine showed only a low antioxidant activity.

### 3.7. Apoptotic Cell Death Induction by Ethanol Extract of *Berberis vulgaris* and Berberine against Tumour Cells

**Figure 6:**
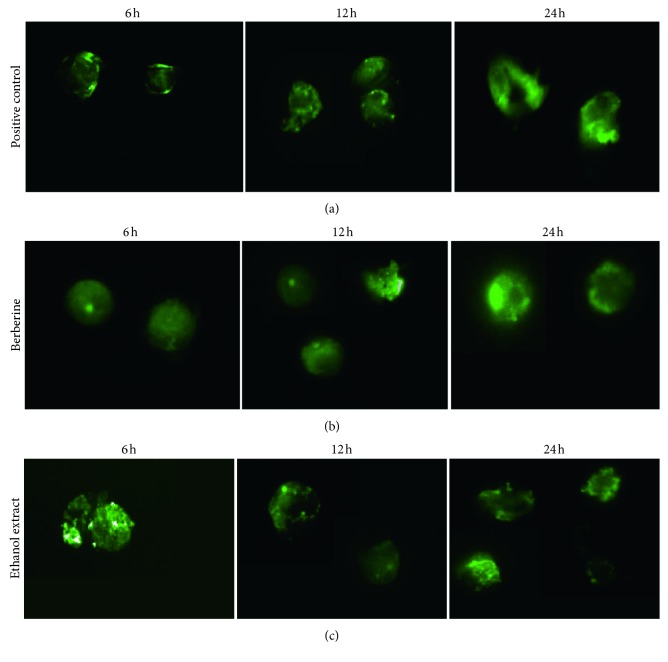
Photographs of annexin V-FITC-stained MCF-7 cells treated with the IC_30_ of berberine and ethanol extract at 6 h, 12 h, and 24 h. (a) Positive control; (b) Berberine; (c) Ethanol extract.

In order to contribute to the understanding of molecular mechanisms involved in the observed cytotoxic activity of *Berberis vulgaris* ethanol extract and its major component, a kinetic study of berberine-induced apoptosis assay was performed using the annexin V-FITC test for 6 h, 12 h, and 24 h. Indeed, utilisation of fluorescein isothiocyanate (FITC)-conjugated annexin V is a standard procedure for monitoring the progression of apoptosis. In fact, early apoptotic cells are annexin V-positive with an intact membrane permeability, whereas late (end-stage) apoptotic cells are annexin V-positive and lose their membrane permeability. Viable cells remained unstained (annexin V-FITC-negative) [14] (Figure 6).

The results showed that berberine and ethanol extract induced MCF-7 cells' killing via apoptosis mechanism. Interestingly, cells treated with berberine are undergoing an early apoptosis unlike the cells treated with ethanol extract that show a late (end-stage) apoptosis at 6 h of stimulation since they are both annexin V-positive with the differences in membrane alteration. Furthermore, over time, berberine and ethanol extract increased the cell membrane alteration as shown at 12 h and 24 h of stimulation (Figure 6).

## 4. Discussion

This study aims to evaluate the antitumour and antioxidant properties of Moroccan *Berberis vulgaris*. The barberry plant was collected in Oujda, east of Morocco, at the end of autumn to early winter (December 2014). We performed the extraction from its root barks using two solvents with different polarities: ethyl acetate and ethanol.

In this work, and for the first time, we evaluated the effect of these two extracts on the human breast adenocarcinoma cell line (MCF-7). Although they exhibited prominent cytotoxic activity against these tumour cells, the ethanol extract was more cytotoxic than the ethyl acetate one with IC_50_ = 3.54 μg/mL and 596.71 μg/mL, respectively. This dissimilarity in their effects is probably due to the different polarities of the two solvents. Furthermore, as the ethanol solvent is more polar than the ethyl acetate one, the phytochemical composition of the ethanol extract has particular molecules that gave it its cytotoxic power against the MCF-7 target cells.

In addition, the phytochemical examination by HPLC/MS performed on the ethanol extract, because of its large cytotoxic activity against the MCF-7 cell line unlike the ethyl acetate extract, revealed the presence of several principal molecules including jatrorrhizine, palmatine, columbamine, berberine, and epiberberine. According to our ethanol extract chromatogram analysis and the results of Ghareeb et al. [3] who found that 1 mg of the ethanol extract of the root barks of *Berberis vulgaris* contains 0.62 mg of berberine, our ethanol extract may be composed mainly of the berberine molecule. These results are in agreement with other findings reporting the presence of these molecules, amongst others depending on the extraction solvent type and the considered part of the plant [26, 27]. Several factors may be responsible for the variation in chemical composition and thus the biological activities of extracts of *Berberis vulgaris* such as climate, soil, cultivation period, and the preservation and extraction methods. Nevertheless, the involvement of minor products in these activities is not to be neglected.

Our results also demonstrated that when comparing the cytotoxic effects of the ethanol extract and berberine, we noted that their effects are similar with nonsignificant differences. This suggests that the cytotoxic effect of the ethanol extract is mainly mediated by berberine, which has a cytotoxic activity with IC_50_ = 8.75 μg/mL against the MCF-7 cells using the MTT test.

Since one of the major problems in cancer chemotherapy is the systemic toxicity due to damaged normal cells by anticancer drugs, we tested our products on normal human peripheral blood mononuclear cells (PBMCs). The results showed that all of the stimuli studied at the inducing toxicity concentrations on the tumour cells have no cytotoxic effect on normal cells. These results correlate with those of Ghareeb et al., who found that the ethanol extract and berberine have no antiproliferative effect on normal PBMCs, although they reported that after incubation of the normal cells for 72 h with ethanol extract or berberine, a slight stimulation of the proliferation of PBMC was observed [3]. This result is very interesting since nontargeting of the tumour cells by anticancer products is the greatest problem causing systemic toxicity. These results corroborate the findings that ethanol extract has an antiviral (especially against hepatitis C) and antifungal effect (against *Aspergillus flavus*), probably due to its immunostimulatory capacity and increasing the activity of phagocytic cells [3] also due to its antioxidant effect for the Malaysian barberry [28].

The tissues and cells exposed to oxidative stress show an alteration of their membrane, DNA damage and reduced repair capacity of the DNA that may contribute to the development of cancer [29]. Thus, the research of powerful antioxidants contributes to the subject of prevention against cancer. In fact, the infusion of *Berberis vulgaris* collected in Peru possesses an interesting antioxidant activity [30]. Similarly, berberine extracted from another barberry plant collected in India has an interesting antioxidant property that gives it its antitumour activity [29]. On the other hand, other investigations found that berberine has an oxidizing activity (production of reactive oxygen-derived ROS) against tumour cells [31–33]. These different results led us to evaluate the antioxidant potential of our extracts and berberine in vitro. Our results showed that ethanol and ethyl acetate extracts have a very large antioxidant potential, and these results confirm the studies of Hanachi and Golkho in reporting the potent antioxidant activity of *Berberis vulgaris* ethanol extract [28].

However, at the same concentrations as the tested extracts, berberine has only 25% inhibition of free radical scavenging compared to both extracts, as measured by the DPPH test, demonstrating that berberine is not very antioxidant. This is in agreement with several reports such as the work of Keawpradub et al. reporting that berberine has a low antioxidant activity (EC_50_ > 100 μg/mL) [34] and the work on the CaSki human cervical cancer line, where Lin et al. found that berberine has an oxidizing activity by increasing the levels of ROS (reactive oxygen derivatives) that destabilizes the potential mitochondrial membrane, thereby dropping cytochrome C in the cytosol that will go into the activation of caspase-3, eventually causing the phenomenon of apoptosis [35]. This is also in agreement with the results of Ho et al., using the human tongue cancer SCC-4 line [36]. In this context, our work suggests that the antioxidant effect of the ethanol and ethyl acetate extracts was due to molecules other than berberine. Furthermore, the results of Hanachi and Golkho showed that the antioxidant effect of the ethanol extract of barberry is probably due to its richness in phenolic compounds [28].

On the other hand, we examined the molecular mechanisms of the cytotoxicity effect of ethanol extract and berberine using annexin-V binding assay at 6 h, 12 h, and 24 h. Phosphatidylserine externalisation was assessed by observing with fluorescence microscopy the extent of streptavidin-fluorescein isothiocyanate (FITC) annexin-V binding. We found that the apoptosis phenomenon starts earlier, at 6 h of stimulation by IC_30_ of both berberine and ethanol extract, noting that the cells treated by ethanol extract showed a damaged membrane unlike the ones treated by berberine at that time. The difference in the membrane shape between the cells treated with ethanol extract and berberine is probably due to other molecules contained in the ethanol extract which have damaged the cell membrane (late apoptosis). Indeed, it has been recently reported that palmatine hydrochloride, one of the ethanol extract compounds, significantly increases the early and late apoptotic rates of the MCF-7 cells [37].

Furthermore, several findings have reported berberine-induced apoptosis in different cell lines, for example, non-small cell human lung cancer, human epidermoid carcinoma cells, A431, U937, B16, HL-60, and MCF-7 cells [38–42].

Berberine treatment could, in a dose-dependent and time-dependent manner, increase Fas protein expression and induce FasL expression in tumour cell lines [43, 44]. It also increases Bax gene protein expression in cancer cells [33, 39, 45–47]. The alteration of berberine on proapoptotic and antiapoptotic gene expressions might be partly mediated by the generation of reactive oxygen species (ROS) [43]. In this work, berberine exhibited apoptosis-promoting and antiproliferative effects in the breast cancer MCF-7 cell line. This finding is in agreement with that of Lin et al. and Patil et al. [42, 48], who reported that the induction of apoptosis by berberine is through cell cycle disruption and DNA fragmentation in a mitochondria-dependent pathway by increasing levels of cytoplasmic cytochrome C, caspase-9 activity, and cleavage of PARP, while decreasing levels of Bcl-2 in MCF-7 cells. It has also been reported that berberine inhibits COX-2 transcriptional activity in a dose-dependent and time-dependent manner in MCF-7 cells [42]. It is possible that berberine may serve as a potential naturally occurring compound for breast cancer therapy.

On the other hand, the evaluation of synergy or antagonism of agents used in combination is an integral part of cancer chemotherapy development. In this paper, we examined the combination effect of berberine combined with S-nitroso-N-acetylpenicillamine (SNAP), a nitric oxide (NO) donor, under physiological conditions. This assay was conducted using the Chou–Talalay method [17]. The choice of NO was based on the fact that this molecule has been described as a potent physiological antitumour agent [23–25]. In addition, NO has been shown to have both protective and deleterious functions [49, 50]. We investigated the effect of NO on the breast cancer cells and found that it exhibited a prominent in vitro cytotoxicity against the MCF-7 cell line in a dose-dependent manner.

To our knowledge, the interaction between berberine and a NO donor has not been previously described in the literature. However, NO can damage cells in many ways involving oxidative stress, DNA damage, protein modification, disruption of energy metabolism, interference with calcium homeostasis, and mitochondrial dysfunction. Depending on the context and severity of the damage, such disturbances may result in cell death either by necrosis or apoptosis, or in a successful repair and cell survival [49]. In our study, berberine, when combined with the NO donor (SNAP used at IC_30_), induced a synergistic cytotoxic activity at concentrations of berberine ranging from 8.40 μM to 33.60 μM. However, at the other tested concentrations, an antagonistic effect was observed. These results may suggest the existence of differential mechanisms of berberine and SNAP interactions in MCF-7 cells in a dose-dependent manner. In fact, preventing or inducing apoptosis by NO may be modulated by dose-dependent interactions; at low concentrations, NO protects cells by inhibiting TNF*α*-induced apoptosis, but at high concentrations, NO induces apoptosis in endothelial cells [10, 50]. This double-edged effect of NO may have significant implications for the interaction with berberine, resulting in the different combination effects (antagonism and synergy) in the breast MCF-7 cancer cells. To our knowledge, this is also the first time where the interaction of berberine and SNAP has been reported.

## 5. Conclusion

In summary, our study demonstrates for the first time the selective cytotoxic effect of the ethanol extract of Moroccan *Berberis vulgaris* against the MCF-7 tumour cell line depending on the dose of exposure without affecting the normal cells and that this cytotoxic effect may be due to its main compound berberine. We also reported that the ethanol extract and berberine display cell lysis by the apoptosis pathway and highlighted their antioxidant actions. On the other hand, this is the first report on the in vitro interaction between berberine and a NO donor.

Our study provides a basis for future clinical studies of berberine in patients with cancer, used alone or in combination with NO-inducer drugs. An adjuvant mechanism-based therapy with berberine compound may significantly improve clinical efficacy. This research, together with the previously reported findings in literature, will help improve our understanding about the molecular mechanisms of berberine as an anticancer agent.

## Figures and Tables

**Table 1:** Summary of the major components of *Berberis vulgaris* ethanol extract.

Molecule number	RT (min)	Molecules	[*M* + *H*]^+^ (*m/z*)	References
*α*	32.93	Jatrorrhizine	338.24	[18, 19]
*β*	15.23	Palmatine	342.23	[20, 21]
*γ*	30.73	Columbamine	338.24	[18, 19]
*δ*	49.31	Berberine	336.22	[18, 19, 22]
*Ɛ*	43.37	Epiberberine	336.26	[19]

**Table 2:** Cytotoxic activity of berberine and SNAP alone against the MCF-7 tumour cell line.

	Berberine					SNAP				
	Concentrations (μM)					Concentrations (μM)				
	0	100	200	400	600	0	100	200	400	600
Lysis (%)	0	43.2 (±0.81)	66.4 (±1.8)	75.81 (±1.3)	81.32 (±0.6)	0	50.9 (±9.8)	58.76 (±10.5)	66.62 (±8.2)	71.22 (±9.4)

**Table 3:** Combination index determination for the combined effect of berberine and NO donor (SNAP).

Berberine (μM)	SNAP (IC_30_ μM)	Fa (B + S)	CI	Description^a^
0.525	15.84	0	14.7	Antagonism
1.050	15.84	0.755	13.8	Antagonism
2.10	15.84	6.410	8.8	Antagonism
4.20	15.84	22.381	2.6	Antagonism
8.40	15.84	39.555	0.7	Synergy
16.80	15.84	49.361	0.5	Synergy
33.60	15.84	52.010	0.6	Synergy
67.20	15.84	52.285	1.2	Antagonism
134.40	15.84	53.103	2.1	Antagonism
268.80	15.84	73.204	1.4	Antagonism
537.60	15.84	80.507	1.1	Antagonism
1075.20	15.84	81.749	1.9	Antagonism

Fa: affected fraction; B: berberine; S: SNAP; B + S: berberine + SNAP; CI: combination index. ^a^CI = 1.00: additive effect; CI < 1.00: synergistic effect; CI > 1.00: antagonistic effect.
